# Male infertility related to an aberrant karyotype, 47,XYY: four case reports

**DOI:** 10.1186/1757-1626-2-28

**Published:** 2009-01-08

**Authors:** Faeza El-Dahtory, Hany M Elsheikha

**Affiliations:** 1 Genetics and Human Reproduction Division, Faculty of Medicine, Mansoura 35516, Egypt; 2 The School of Veterinary Medicine and Science, The University of Nottingham, Sutton Bonington Campus, Loughborough, Leicestershire, LE12 5RD, UK

## Abstract

**Background:**

47,XYY syndrome is a sex chromosomal abnormality observed in humans, with a prevalence of 0.1% of male births. Sex chromosome anomalies are more frequently associated with male infertility.

**Case report:**

We present here four cases of infertile men with azoospermia or severe oligozoospermia attending a genetic and fertility clinic. Chromosomal analysis of the peripheral blood lymphocytes demonstrated the constitutional karyotype of 47, XYY. Using fluorescence in situ hybridization (FISH) the presence of extra Y chromosome was confirmed, supporting the cytogenetic finding.

**Conclusion:**

The 47,XYY syndrome is relatively uncommon and can be missed clinically because of its variable clinical presentations. Accurate diagnosis of this constitutional karyotype provides a valuable aid in the counselling and early management of the patients who undertake fertility evaluation.

## Background

The XYY syndrome is an aneuploidy of the sex chromosomes in which a human male receives an extra Y chromosome, producing a 47,XYY karyotype. This chromosomal anomaly occurs in one of 1,000 live male births in the general population, but more frequent in infertile populations [1]. In the majority of cases the phenotypic features remain normal, which hampers further management based on the specific requirements associated with a specific etiology. However, 47,XYY boys have an increased risk of behavior problems, increased growth velocity during earliest childhood, with tall stature, mild learning disability, and delayed speech and language skills [2]. We herein report four cases showing XYY syndrome out of 132 infertile male subjects investigated.

## Methods

### Patients

The four cases were selected from 132 infertile men attending the genetic and fertility clinic at the academic hospital, Faculty of Medicine, Mansoura University, Egypt and were identified by a clinical geneticist as possibly carrying a chromosomal abnormality. The patients were interviewed about their histories and their reproductive problems, family background, and possible consanguinity. The interview was followed by a physical examination in order to identify anatomical problems. The urethral fluid and semen were tested for microbial infections. Hormonal profile using quantitative analysis of FHS, LH and testosterone was carried out in all patients.

### Conventional cytogenetic analysis

Blood samples were collected from all patients into heparinised test tubes. Peripheral blood samples of the patient were cultured for 72 hours in RPMI-1640 medium supplemented with fetal bovine serum and phytohemagglutinin. Cytogenetic analysis was performed in all patients by using the GTG banding technique [3]. At least 30 metaphases were analysed. The best metaphases were photographed to determine the patients' karyotype. The karyotypes were described according to the ISCN 95 nomenclature [4].

### FISH

We performed FISH using fluorescence-labeled X- and Y- centromeric probes according to the manufacturer's recommendations. Analysis was done using a Zeiss Axioplan epifluorescent microscope equipped with appropriate filters. Images were captured using Cytovision V2.81 Image Analysis software (Applied Imaging International, San Jose, CA, USA).

## Results

### Case presentation

#### Case 1

A 31-year-old man was admitted to the hospital because of sterility. The patient was the oldest of three normal siblings born to consanguineous healthy parents. He had normal phenotypic features with unremarkable medical history. He had tall stature (height, 193 cm; weight, 74 kg) with an arm span of 206 cm. Endocrinological testing demonstrated increase in the FSH and LH levels (26.5 and 16.9 mIU/mL, respectively), and a very low level of testosterone (1.8 ng/mL). Seminal analysis revealed azoospermia.

#### Case 2

A 30-year-old man visited our outpatient clinic complaining of infertility of 4 years' duration. There was no family history of similarly affected members. The parents were cousins. Physical examination revealed a normal male with a height of 187 cm and a weight of 71 kg. Both testicular volumes were 14 ml. Measurement of serum hormone levels demonstrated normal values for LH (4.2 mIU/mL) and testosterone (6.8 ng/mL) with elevated level of FSH (21.3 mIU/mL). Seminal analyses revealed severe oligozoospermia with a sperm concentration of 0.7 × 10^6^/ml (4.9 × 10^6^/ejaculate), 24% motility, and 5% normal morphology.

#### Case 3

A 30-year-old man with 4 years of primary infertility due to oligozoospermia 1.3 × 10^6^/ml (8.5 × 10^6^/ejaculate), 18% normal motility and 4% normal morphology. He is the first boy born to non consanguineous parents with two normal brothers. No family history of similarly affected members. Blood work results included high FSH and LH, (19.3 and 17.3 mIU/ml, respectively), and low testosterone (2.8 ng/ml).

#### Case 4

A 40-year-old man presented with 3 years of primary infertility. He is the youngest boy born of non consanguineous marriage with two normal brothers. There were no relevant findings regarding his past history and habits. The family history was unremarkable. Results on physical examination and ultrasound analysis of his genitourinary system were normal, with normal testicular volume. Results on hematologic and biochemical parameters were normal. Levels of FSH and LH were high (14.5 and 18.6 mIU/ml, respectively) and testosterone level was within low normal limit (3.6 ng/ml). Two semen analyses demonstrated oligozoospermia with a sperm count of 2.5 × 10^6^/ml (8 × 10^6^/ejaculate), 25% normal motility and 4% normal morphology.

### Cytogenetic features

**Figure 1:**
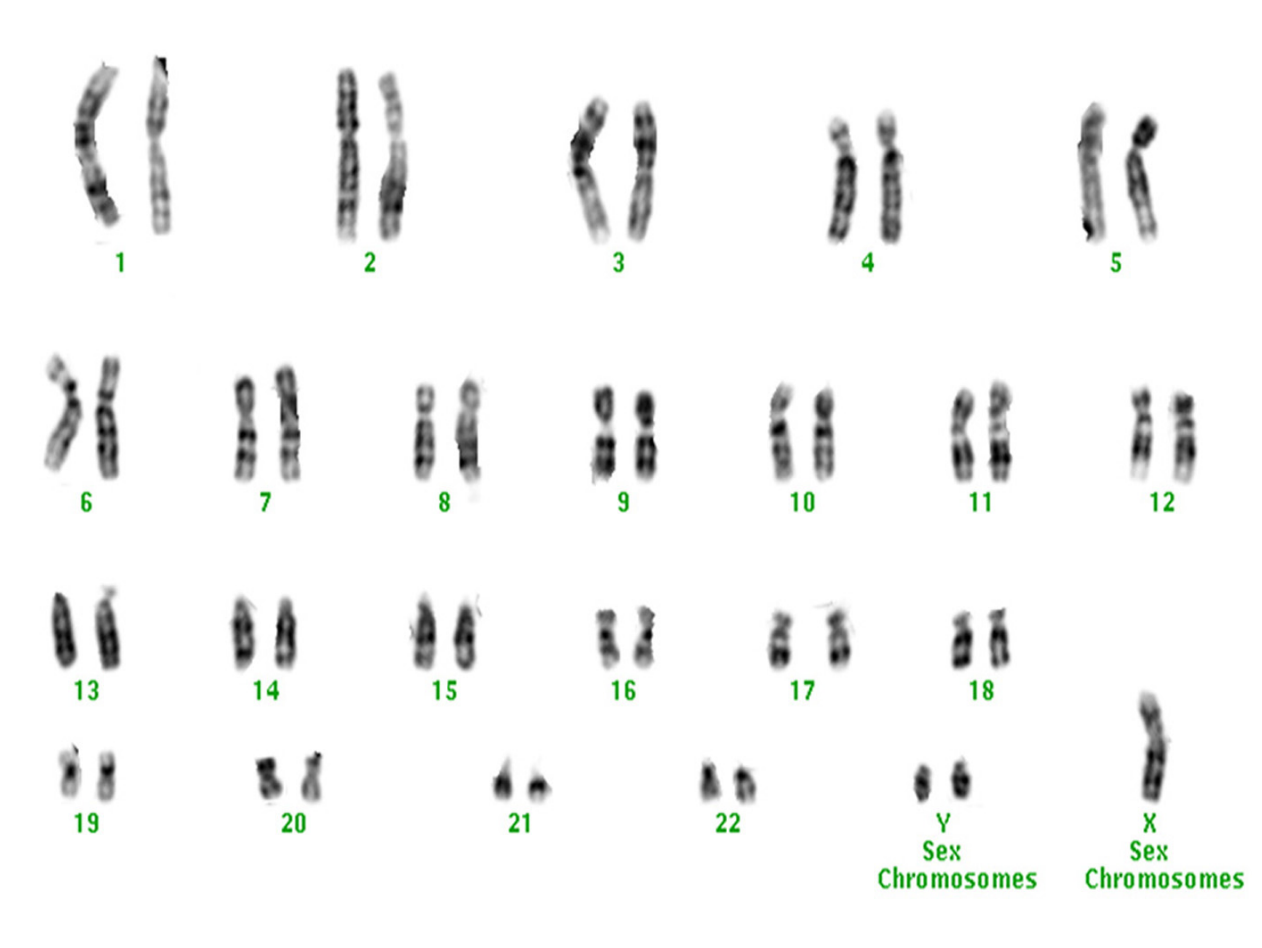
**Karyogram of males with 47,XYY syndrome**.

**Figure 2:**
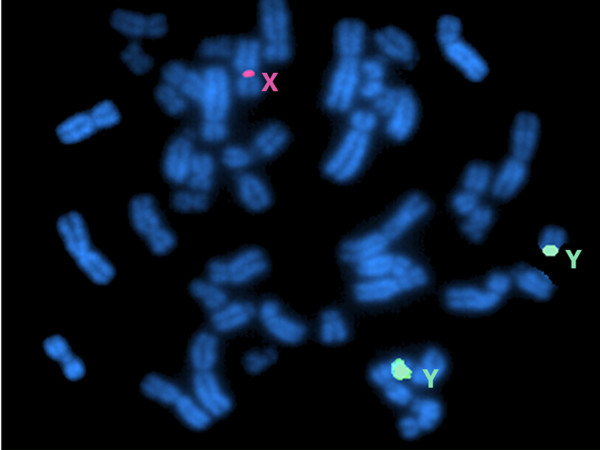
**Metaphase FISH analysis showing two green signals of the centromer of the Y chromosomes and one red signal of the centromer of the X chromosome confirming the karyotype of XYY**.

**Figure 3:**
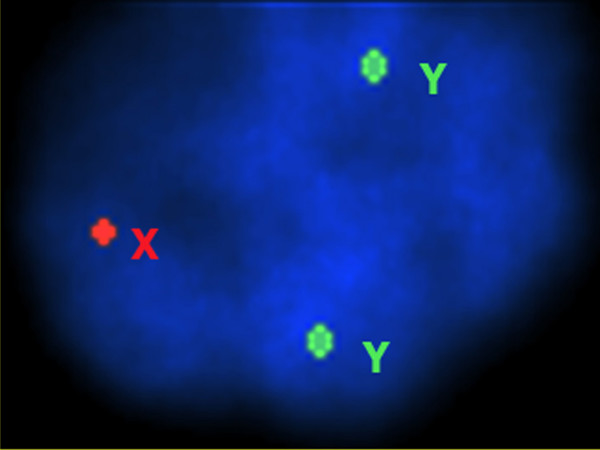
**Interphase FISH analysis showing two green signals of the Y chromosomes and one red signal of the X chromosome confirming the karyotype of XYY**.

Chromosomes studies with conventional cytogenetic analysis revealed numerical chromosomal abnormalities in the four patients. The GTG-banding revealed the abnormal non-mosaic 47,XYY karyotype in metaphase cells in all four patients (Fig. 1). The presence of extra Y chromosome was confirmed by FISH analysis, using fluorescence-labeled X- and Y- centromeric probes (Figs. 2 and 3).

## Discussion and conclusion

The existence of a genetic component to human infertility has been suggested, although neither the specific abnormalities involved, nor their genetic mechanism of transmission, are completely defined. The present report aimed to present four cases of male patients who admitted to the genetic and fertility unit at Mansoura University Hospital because of infertility problems. Cytogenetic analysis using GTG-banding technique revealed the karyotype 47,XYY among all patients. Although cytogenetic analyses are sometimes tedious, they are very important for the identification of a variety of syndromes. Additionally, the success of providing a clinical diagnosis with cytogenetic techniques can be improved using FISH and other complementary molecular biology techniques. The latter coupled with conventional karyotying, can largely overcome the limitations of conventional banding in the accurate diagnosis and interpretation of subtle or complex chromosomal abnormalities. With both approaches, a definitive diagnosis of a 47,XYY chromosomal disorder was achieved in our study. The information obtained by such techniques provides a basis for deciding the necessary clinical management and genetic counselling of patients who require this service.

The medical history of all patients was unremarkable. There was no familial history of congenital malformation nor of exposure to drugs or toxic environmental agents. Routine analyses showed no infectious cause for their infertility. Testosterone levels are normal in 47,XYY males [5], but patients in our study had low to normal testosterone level and high to normal levels of FSH and LH. Semen analysis showed abnormal profile. Semen abnormalities may result from multiple causes such as Y chromosome microdeletions and CFTR gene mutations [6,7], but none of these was found in any of the four patients.

It was hypothesised that human male subfertility may have a familial component [8]. From the data presented, it appears that infertility may have a genetic basis since all the four patients came from families with consanguinity marriage, but it was out of the scope of this report to evaluate the relationship between consanguinity and this chromosomal anomaly or the possibility that consanguinity may influence the phenotypic characteristics. In general, 47,XYY is not inherited, but usually occurs as a random error in chromosome separation during the formation of sperm cells, leading to formation of sperm cells with an extra copy of the Y chromosome. If one of these peculiar sperm cells contributes to the genetic makeup of a child, the child will have an extra Y chromosome in each of the body's cells [9]. In some cases, the addition of an extra Y chromosome results from nondisjunction during cell division during a post-zygotic mitosis in early embryonic development. This can produce 46,XY/47,XYY mosaics [9].

Cytogenetic abnormalities have been known to be important causes of male infertility for decades and chromosomal abnormalities are more frequently observed in the population of azoo- and/or oligozoospermic males than in the general population. Men with a 47,XYY karyotype are generally fertile and there is no evidence of transmission of the extra Y chromosome to their progeny because the supernumerary Y-chromosome is eliminated during meiosis; and some XYY germ cells can complete meiosis and produce mature aneuploid sperm [10]. However, they are seen more frequently in infertile populations. In this report all patients with 47,XYY karyotype are infertile. However, generalizations cannot be made regarding the correlation between karyotype and infertility phenotype. This finding may be coincidental. No systematic studies have been published showing that the XYY syndrome is associated with increased frequency of infertility. Only a few case reports of abnormalities of fertility have been published in males with the XYY syndrome [11]. Nonetheless, the relatively high prevalence of this abnormal karyotype in infertile men in our report justifies the use of karyotyping to evaluate males with reproductive abnormalities especially in the case of those with high degrees of consanguinity.

## Abbreviations

FSH: follicle stimulating hormone; FISH: fluorescence in situ hybridization; GTG: G-banded using trypsin and Giemsa; LH: luteinising hormone

## Consent

Written informed consent was obtained from the patients for publication of this report.

## Competing interests

The authors declare that they have no competing interests.

## Authors' contributions

FED performed cytogenetic analysis and acquisition of data. HE contributed to the analysis, review of literature and wrote the paper. Both authors read and approved the final manuscript.

## References

[B1] Martin RH Cytogenetic determinants of male fertility Hum Reprod 2008 14 379 390 10.1093/humupd/dmn017 PMC2423221 18535003

[B2] Ratcliffe SG Butler GE James M Evans JA, Hamerton JL, Robinson A Edinburgh study of growth and development of children with sex chromosome abnormalities Children and Young Adults with Sex Chromosome Aneuploidy 1990 New York: Wiley-Liss for the National Foundation-March of Dimes 59 115

[B3] Moorhead PS Nowell PC Mellman WJ Battips DM Hungerford D Chromosome preparations of leucocytes cultured from human peripheral blood Exp Cell Res 1960 20 613 616 10.1016/0014-4827(60)90138-5 13772379

[B4] ISCN 95 Mitelman F (ed) An International System for Human Cytogenetic Nomenclature 1995 Karger, Basel

[B5] Ratcliffe SG Read G Pan H Fear C Lindenbaum R Crossley J Prenatal testosterone levels in XXY and XYY males Horm Res 1994 42 106 109 10.1159/000184157 7995613

[B6] Foresta C Moro E Ferlin A Y chromosome microdeletions and alterations of spermatogenesis Endocr Rev 2001 22 226 239 10.1210/er.22.2.226 11294825

[B7] Cruger DG Agerholm I Byriel L Fedder J Bruun-Petersen G Genetic analysis of males from intracytoplasmic sperm injection couples Clin Genet 2003 64 198 203 10.1034/j.1399-0004.2003.00128.x 12919133

[B8] Lilford R Jones AM Bishop DT Thornton J Mueller R Case-control study of whether subfertility is familial Br Med J 1994 309 6954 570 573 8086942 10.1136/bmj.309.6954.570 PMC2541440

[B9] Robinson DO Jacobs PA The origin of the extra Y chromosome in males with a 47,XYY karyotype Hum Mol Genet 1999 8 2205 2209 10.1093/hmg/8.12.2205 10545600

[B10] Morel F Roux C Bresson JL Sex chromosome aneuploidies in sperm of 47,XYY men Arch Androl 1999 43 27 36 10.1080/014850199262706 10445102

[B11] Rives N Milazzo JP Miraux L North MO Sibert L Macé B From spermatocytes to spermatozoa in an infertile XYY male Int J Androl 2005 28 304 310 10.1111/j.1365-2605.2005.00540.x 16128991

